# MicroRNA-381 Regulates Proliferation and Differentiation of Caprine Skeletal Muscle Satellite Cells by Targeting *PTEN* and *JAG2*

**DOI:** 10.3390/ijms232113587

**Published:** 2022-11-05

**Authors:** Jiyuan Shen, Jiqing Wang, Huimin Zhen, Yan Liu, Lu Li, Yuzhu Luo, Jiang Hu, Xiu Liu, Shaobin Li, Zhiyun Hao, Mingna Li, Zhidong Zhao

**Affiliations:** Gansu Key Laboratory of Herbivorous Animal Biotechnology, College of Animal Science and Technology, Gansu Agricultural University, Lanzhou 730070, China

**Keywords:** miR-381, caprine skeletal muscle satellite cells, viability, proliferation, differentiation

## Abstract

In our previous study, microRNA (miR)-381 was found to be the most down-regulated miRNA in skeletal muscle of Liaoning cashmere goats with higher skeletal muscle mass, but the molecular mechanism involved remains unclear. In this study, primary caprine skeletal muscle satellite cells (SMSCs) were isolated and identified. We investigated the effect of miR-381 on the viability, proliferation and differentiation of caprine SMSCs, and the target relationships of miR-381 with jagged canonical Notch ligand 2 (*JAG2*) and phosphatase and tensin homolog (*PTEN*). Cells isolated were positive for SMSC-specific marker protein Pax7. This suggests that purified SMSCs were obtained. The expression level of miR-381 achieved a peak value on day 4 after SMSC differentiation, and miR-381 also significantly increased the expression levels of myogenic differentiation marker genes: myosin heavy chain (*MyHC*), myogenin (*MyoG*) and myocyte enhancer factor 2C (*MEF2C*) in differentiated SMSCs, the area of MyHC-positive myotubes and the myogenic index. These findings suggest that miR-381 promoted myogenic differentiation of caprine SMSCs. The CCK8 assay and EDU staining analysis showed that miR-381 mimic both inhibited the viability of SMSCs and decreased the percentage of EDU-labeled positive SMSCs. In contrast, miR-381 inhibitor had the opposite effect with miR-381 mimic. A dual luciferase reporter assay verified that miR-381 can target *JAG2* and *PTEN* by binding to the 3′-untranslated regions (3′-UTR) of the genes. The transfection of miR-381 mimic into caprine SMSCs resulted in decreases in expression levels of *JAG2* and *PTEN*, while miR-381 inhibitor increased the two target genes in expression. This is the first study to reveal the biological mechanisms by which miR-381 regulates caprine SMSC activities.

## 1. Introduction

Skeletal muscle is considered the most economically valuable tissue of meat-producing livestock. The growth and development of skeletal muscle directly affect meat yield and quality, naturally determining the commercial return of goat producers. Skeletal muscle satellite cells (SMSCs) are myogenic stem cells of postnatal muscle, and their activity, proliferation and differentiation play crucial roles in supporting muscle growth, hypertrophy and regeneration of postnatal skeletal muscle [1]. In this context, an in-depth understanding of the biological mechanisms that regulate proliferation and differentiation of SMSCs provides an opportunity to improve meat yield and quality in goats. Studies of SMSCs in domestic animals have mainly been concentrated on cattle [2], pigs [3] and chicken [4]. These studies revealed that numerous genes and non-coding RNAs are involved in the regulation of proliferation and differentiation of SMSCs.

MicroRNAs (miRNAs) are a class of small, evolutionarily conserved non-coding RNA molecules (~22 nucleotides). They can inhibit translation or promote the degradation of the target mRNAs at a post-transcriptional level by complementarily binding to the 3′-untranslated regions (3′-UTR) of the target genes [5]. Previous studies have suggested that miRNAs have important roles in regulating the development of SMSCs. For example, miR-199b and miR-34c repressed porcine SMSC proliferation by targeting jagged canonical Notch ligand 1 (*JAG1*) and Notch receptor 1 (*NOTCH1*), respectively [3,6]. miR-128 has been reported to regulate the proliferation and differentiation of bovine SMSCs by repressing the expression of Sp1 transcription factor (*SP1*) [7]. In addition, miR-21-5p, miR-27b and miR-1 also affect SMSC activity in chicken [4], sheep [8] and goats [9], respectively.

In our previous study, miR-381 was found to be the most down-regulated miRNA in skeletal muscle of Liaoning cashmere goats with higher meat yield and larger muscle fiber size, when compared to Ziwuling black goats [10]. For example, the carcass weight of Liaoning cashmere goats was 14.10 ± 1.17 kg, which was higher than that of Ziwuling black goats with a carcass weight of 7.45 ± 1.28 kg. The diameter and cross-sectional area of muscle fibers from Liaoning cashmere goats were 38.52 ± 2.20 μm and 1902.91 ± 156.92 μm^2^, respectively, which was larger than Ziwuling black goats with a diameter of 29.09 ± 3.81 μm and a cross-sectional area of 1117.72 ± 210.10 μm^2^. Meanwhile, miR-381 has also been reported to be differentially expressed in muscle tissues between different developmental stages of Anhui white goats and Jianzhou Da’er goats [11,12]. This suggests that miR-381 may play an important role in the growth and development of caprine skeletal muscle. However, there have been no reports on the function of miR-381 in the development of SMSCs. In this study, we investigated the effect of miR-381 on the proliferation and differentiation of caprine SMSCs. We also verified the target relationships of miR-381 with *JAG2* and phosphatase and tensin homolog (*PTEN*).

## 2. Results

### 2.1. Isolation, Identification and Myogenic Differentiation of Caprine SMSCs

After purification three times using the differential adhesion method, cells began to grow adherently after culture for 48 h (Figure S1A). At 96 h after culture, the volume of cells became large and the morphology tended to be stable, with a predominantly spindle shape or fusiform shape (Figure S1B). The immunofluorescence analysis indicated that the cells isolated were positive for SMSC-specific marker protein Paired Box 7 (Pax7) (Figure S1C). The results suggest that purified SMSCs were obtained in the study. Additionally, isolated SMSCs can differentiate into myotubes on day 6 of differentiation initiation (Figure S1D), suggesting that isolated SMSCs had superior ability of myogenic differentiation.

### 2.2. Expression Profiles of MyoG, MyHC and miR-381 during Myogenic Differentiation

**Figure 1:**
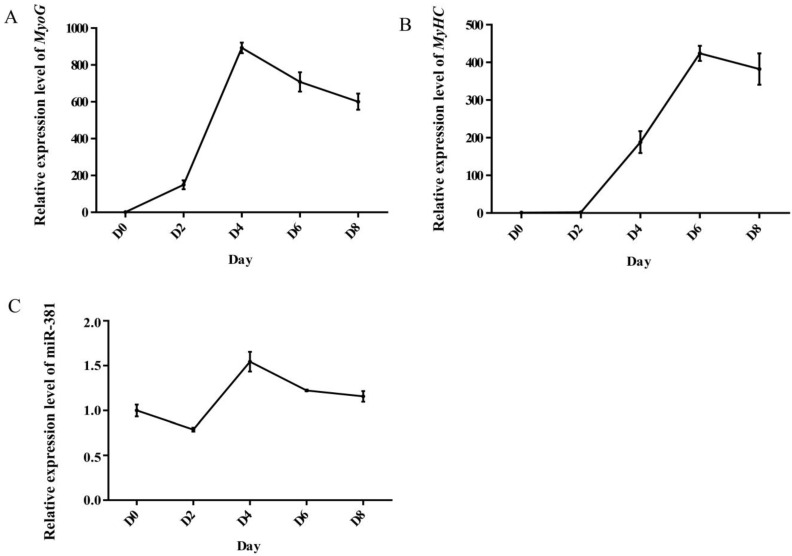
Expression level of *MyoG* (**A**), *MyHC* (**B**) and miR-381 (**C**) at different differentiation stages of caprine skeletal muscle satellite cells (SMSCs).

Myogenin (*MyoG*) and myosin heavy chain (*MyHC*) are important myogenic differentiation marker genes and their expression levels were therefore detected during myogenic differentiation. The expression level of *MyoG* gradually increased from day 0 to day 4 after differentiation and then decreased (Figure 1A), while the expression of *MyHC* achieved a peak value on day 6 after differentiation (Figure 1B). The results suggest that caprine SMSCs differentiated normally and fully and can be used for further investigation. Similarly, the expression level of miR-381 also achieved a peak value on day 4 after differentiation (Figure 1C). The result indicates that miR-381 may play a role in myogenic differentiation.

### 2.3. miR-381 Accelerates Myogenic Differentiation of Caprine SMSCs

**Figure 2:**
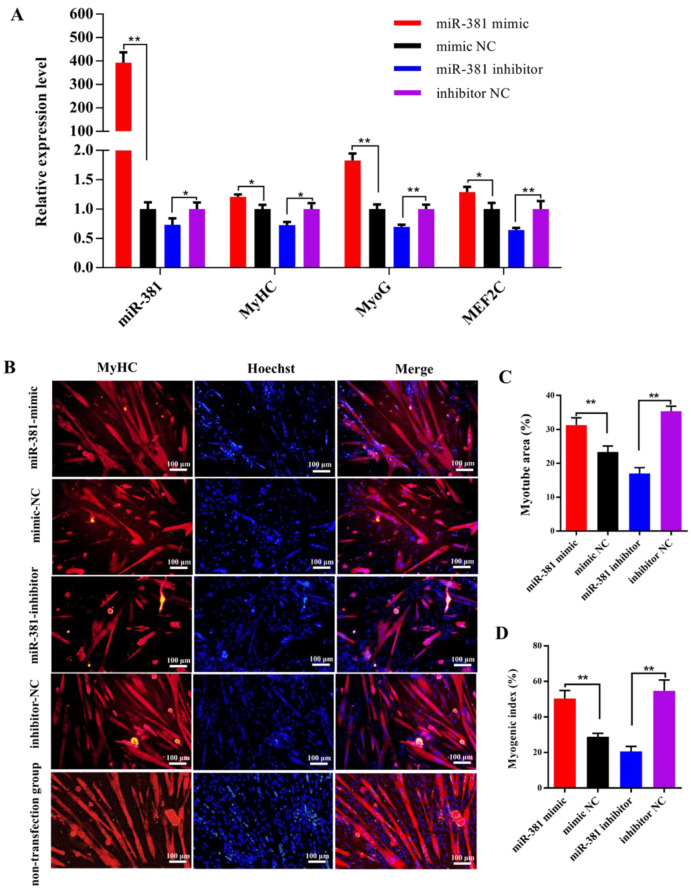
The miR-381 promoted differentiation of caprine skeletal muscle satellite cells (SMSCs). (**A**) Expression levels of miR-381 and myogenic differentiation marker genes *MyHC*, *MyoG* and *MEF2C* in differentiated caprine SMSCs transfected with miR-381 mimic, miR-381 inhibitor and their corresponding negative control (NC) groups. (**B**) The effect of miR-381 on the formation of MyHC-positive myotubes detected by anti-MyHC immunofluorescence staining. Relative myotube area (**C**) and myogenic index (**D**) of differentiated caprine SMSCs counted by ImageJ software v1.8.0 (NIH, Bethesda, MD, USA). * *p* < 0.05 and ** *p* < 0.01.

It was found from reverse transcription–quantitative PCR (RT-qPCR) analysis that over-expression of miR-381 significantly increased the expression levels of miR-381 and myogenic differentiation marker genes *MyHC* and *MyoG* and myocyte enhancer factor 2C (*MEF2C*) in differentiated SMSCs, while inhibition of miR-381 decreased their expression levels (Figure 2A, *p* < 0.05). Immunofluorescence analysis results showed that over-expression of miR-381 promoted the formation of MyHC-positive myotubes (Figure 2B,C) and increased the myogenic index (Figure 2D; *p* < 0.01), whereas decreased MyHC-positive myotube area and myogenic index were observed when the expression of miR-381 was inhibited (Figure 2B–D; *p* < 0.01). Meanwhile, the SMSCs with non-transfection of anything exhibited normal differentiation status (Figure 2B). The results suggest that miR-381 promoted myogenic differentiation of caprine SMSCs by up-regulating the expression of myogenic differentiation marker genes *MyHC*, *MyoG* and *MEF2C*.

### 2.4. miR-381 Suppresses Viability and Proliferation of Caprine SMSCs

**Figure 3:**
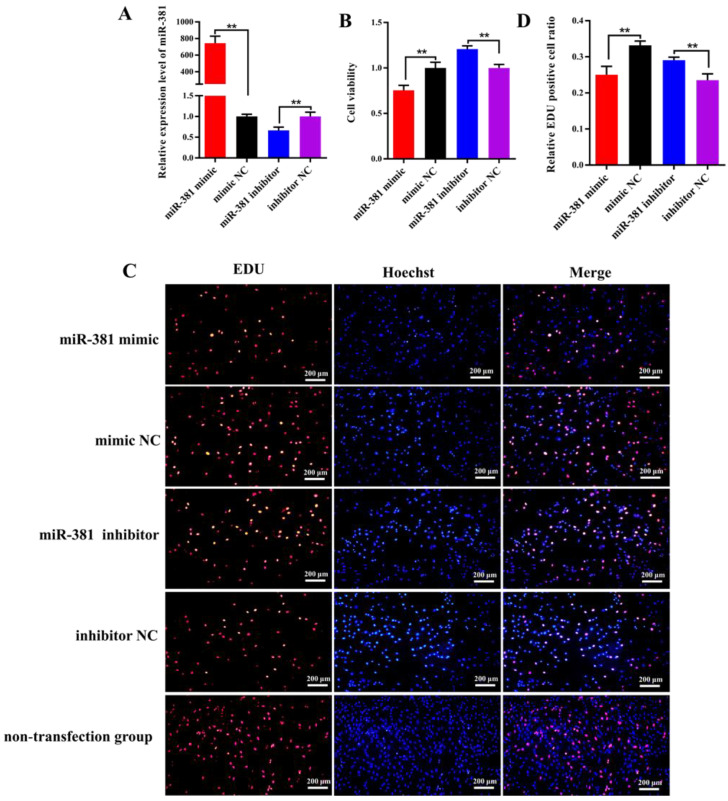
The miR-381 suppressed the viability and proliferation of caprine skeletal muscle satellite cells (SMSCs). (**A**) Transfection efficiency detection of miR-381 mimic and miR-381 inhibitor. (**B**) The effect of miR-381 on the viability of caprine SMSCs when miR-381 mimic and miR-381 inhibitor were transfected into SMSCs. (**C**) The effect of miR-381 on the proliferation of SMSCs detected using EDU assay. EDU, Hoechst and Merge represent the number of EDU-positive SMSCs, the total number of SMSCs and the proportion of EDU-positive SMSCs in the total SMSCs, respectively. (**D**) The percentage of EDU-positive SMSCs counted by ImageJ software. ** *p* < 0.01.

The RT-qPCR analysis results revealed that the expression level of miR-381 in SMSCs transfected with miR-381 mimic was 745-fold higher than that in the mimic NC group (Figure 3A; *p* < 0.01). On the contrary, the expression of miR-381 had a significant decrease in SMSCs transfected with miR-381 inhibitor compared with the inhibitor NC group (Figure 3A; *p* < 0.01). This indicates that the miR-381 mimic and miR-381 inhibitor were transfected into SMSCs successfully. The CCK8 assay and EDU staining analysis showed that over-expression of miR-381 markedly inhibited the viability of SMSCs (Figure 3B, *p* < 0.01) and decreased the percentage of EDU-positive SMSCs (Figure 3C,D; *p* < 0.01), while miR-381 inhibitor increased the viability of SMSCs and the percentage of EDU-positive SMSCs (Figure 3B–D; *p* < 0.01). Meanwhile, the SMSCs with non-transfection of anything also exhibited normal growth status in EDU staining assay (Figure 3C).

**Figure 4:**
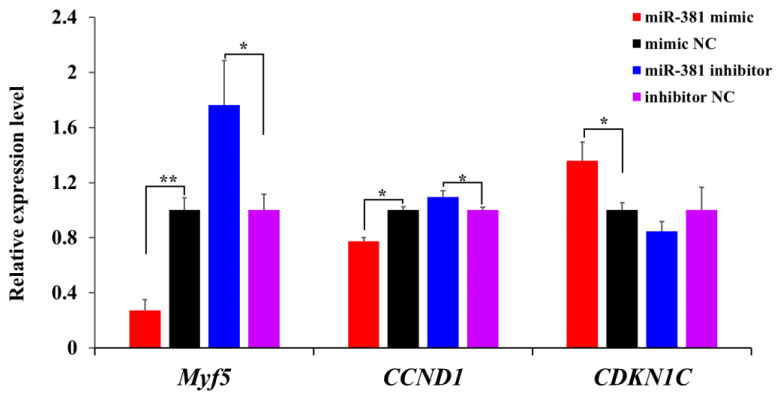
The effect of miR-381 on the expression levels of three proliferation marker genes *Myf5*, *CCND1* and *CDKN1C* in caprine SMSCs detected using RT-qPCR. * *p* < 0.05, ** *p* < 0.01.

To further evaluate the effect of miR-381 on SMSC proliferation, the expression levels of three proliferation marker genes were measured in SMSCs transfected with miR-381 mimic and miR-381 inhibitor. The results showed that the expression levels of myogenic factor 5 (*Myf5*) and cyclin D1 (*CCND1*) were decreased in caprine SMSCs transfected with miR-381 mimic compared to the mimic NC group, while cyclin-dependent kinase inhibitor 1C (*CDKN1C*) was increased (Figure 4, *p* < 0.05). The opposite effect of miR-381 on the expression levels of the three genes was also observed when the expression of miR-381 in SMSCs was inhibited (Figure 4, *p* < 0.05). Taken together, these results suggest that miR-381 inhibited the viability and proliferation of caprine SMSCs.

### 2.5. Prediction and KEGG Pathway Analysis of the Target Genes of miR-381

**Figure 5:**
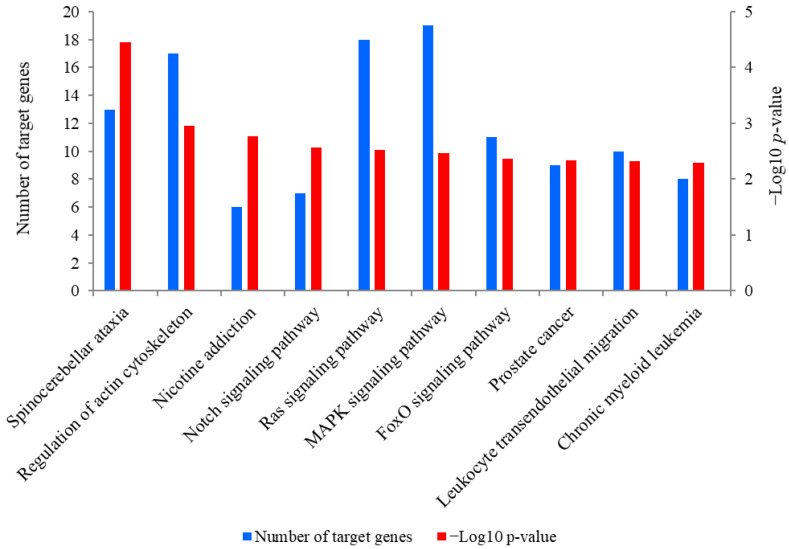
The top 10 KEGG pathways enriched for predicted target genes of miR-381. The left-hand Y-axis represents the number of the target genes of miR-381 involved in a pathway, while the Y axis on the right side shows the value of −Log10 (*p*-value).

A total of 889 target genes were predicted for miR-381. The Kyoto Encyclopedia of Genes and Genomes (KEGG) analysis revealed that these target genes were mainly enriched in spinocerebellar ataxia, regulation of actin cytoskeleton, nicotine addiction and the Notch signaling pathway (Figure 5). Of these pathways, the Notch signaling pathway was closely associated with SMSC activities and it was also significantly enriched by seven target genes of miR-381, including *JAG2*, ataxin 1 like (*ATXN1L*), ataxin 1 (*ATXN1*), aph-1 homolog B, gamma-secretase subunit (*APH1B*), hes related family bHLH transcription factor with YRPW motif 1 (*HEY1*), recombination signal binding protein for immunoglobulin kappa J region (*RBPJ*) and lysine acetyltransferase 2B (*KAT2B*). Meanwhile, *PTEN* is a crucial upstream regulator of the Notch signaling pathway and was also a potential target gene of miR-381. The genes *JAG2* and *PTEN* were therefore selected for the dual luciferase reporter assay.

### 2.6. Validation of Target Relationships of miR-381 with the Target Genes

**Figure 6:**
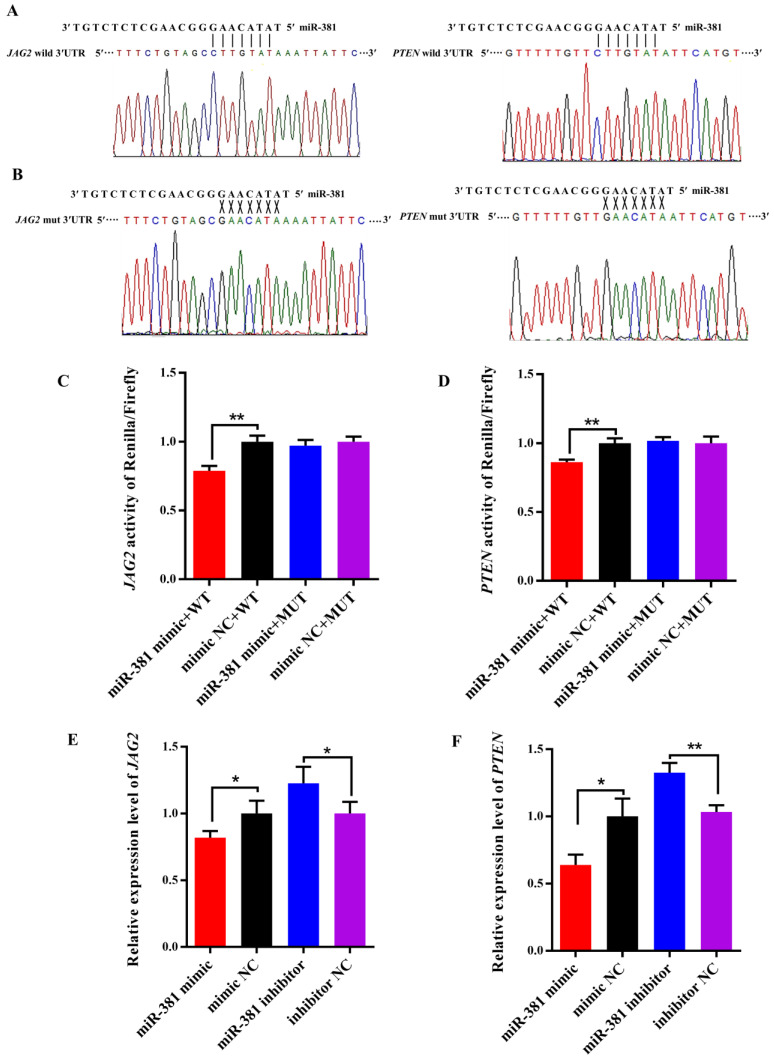
Validation of predicted target genes of miR-381 using dual luciferase reporter assay. (**A**) The binding sequences of miR-381 with the 3′UTR of *JAG2* and *PTEN* in wild-type dual luciferase reporter vectors. (**B**) The complementary sequences of the binding sequences in mutant-type dual luciferase reporter vectors. (**C**,**D**) The luciferase activities of the target genes *JAG2* and *PTEN* for miR-381 detected by dual luciferase reporter assay. (**E**,**F**) The effect of miR-381 on the expression levels of *JAG2* and *PTEN.* * *p* < 0.05 and ** *p* < 0.01.

As shown in Figure 6A, there were potential binding sites in the 3′UTR of both *JAG2* and *PTEN* for miR-381 in wild-type pmiR-RB-Report™ vectors. However, the complementarily sequences of the binding sites were found in mutant-type pmiR-RB-Report™ vectors (Figure 6B). The dual luciferase reporter assay revealed that over-expression of miR-381 significantly decreased luciferase activities of *JAG2* and *PTEN* in HEK293 T cells transfected with wild-type pmiR-RB-Report™ vector when compared to the NC group (Figure 6C,D; *p* < 0.01). However, there were no changes in luciferase activities of the two genes in HEK293 T cells co-transfected with corresponding mutant reporter vector and miR-381 mimic when comparing to its NC group (Figure 6C,D; *p* > 0.05). These suggest that miR-381 targets *JAG2* and *PTEN* by binding to their 3′UTR. Meanwhile, over-expression of miR-381 also decreased the expression levels of *JAG2* and *PTEN* in caprine SMSCs, while inhibition of miR-381 increased their expression levels (Figure 6E,F; *p* < 0.05).

## 3. Discussion

Postnatal muscle growth of animals is mainly due to hypertrophy of muscle fiber, particularly increases in muscle fiber size. SMSCs are myogenic stem cells that can provide new nuclei to support hypertrophy and regeneration of postnatal muscle through synthesizing muscle protein [13]. In this context, SMSCs have been widely used as models for investigating the growth and development of skeletal muscle in various species [3,4,6,7]. In our previous study, miR-381 was identified as the most down-regulated miRNA (fold change = 20.0, *p* = 1.6 × 10^−8^) in Liaoning cashmere goats with higher muscle fiber size and carcass weight compared to Ziwuling black goats [10]. It was therefore inferred that miR-381 may regulate the size of caprine muscle fiber by regulating the activities of SMSCs.

To validate our hypothesis, primary caprine SMSCs were first isolated from postnatal muscle of a Ziwuling black goat. Pax7 is a specific marker protein of SMSCs, and has been widely used to identify the purity of SMSCs in multiple species, including goats [14], pigs [15] and sheep [16]. In this study, isolated cells were found to be positive for Pax7 protein, suggesting that purified caprine SMSCs were obtained in this study.

SMSCs have the superior ability of myogenic differentiation, in that they can produce multinucleated myotubes by a process of proliferation and differentiation. MyHC is a marker protein in the late stage of myogenic differentiation of SMSCs, and is generally also positive for myotubes. In this context, MyHC has been widely used to identify myotubes formed during myogenic differentiation of SMSCs in various species [4,6,9]. In this study, MyHC-positive myotubes formed on day 6 after myogenic differentiation inducement (Figure 2B). In addition to MyHC, myogenic differentiation was also positively regulated by other myogenic regulators, such as MyoG and MEF2C, which belong to myogenic regulatory factors (MRFs) and the MEF2 family, respectively. The two families have been used as markers of muscle cell differentiation [17]. In this study, it was found that the expression levels of *MyoG* and *MyHC* exhibited the tendency of increasing first and then decreasing with myogenic differentiation of SMSCs. Similar expression patterns of the genes have also been found in myogenic differentiation of SMSCs isolated from Nanjiang brown goats [18]. This suggests that the differentiation process of caprine SMSCs isolated in the study occurred as normal.

In this study, to eliminate the experimental error as soon as possible, negative controls for miR-381 mimic and miR-381 inhibitor were designed. Meanwhile, the cells without transfection of anything were designated as the non-transfection group to indicate the normal growth status of the SMSCs in myogenic differentiation and proliferation assay. It was notable that miR-381 increased the expression levels of three myogenic differentiation marker genes, the area of MyHC-positive myotubes and the myogenic index. These findings suggest that miR-381 promoted the differentiation of caprine SMSCs. Given the roles of *MyHC*, *MyoG* and *MEF2C* in myogenic differentiation, it was inferred that miR-381 may regulate caprine SMSC differentiation by regulating the expression of the three genes. However, this speculation needs to be further verified. At present, to the best of our knowledge, there have been no reports about the role of miR-381 in myogenic differentiation in other animals. However, miR-381 has been reported to promote the differentiation of other types of cells, including cattle preadipocytes [19], mouse embryonic palatal mesenchymal cells [20] and human retinal progenitor cells [21]. These studies further support our findings.

CCK-8 is a method for detecting the viability of cells. It has higher detection sensitivity than other methods such as MTT, XTT, MTS, WST-1 and the colorimetric method [22]. EDU assay can precisely detect DNA replication occurring during the S-phase of cells [23]. The two methods have been widely used for viability and proliferation analyses of various cells. In our study, miR-381 was found to inhibit the viability and proliferation of caprine SMSCs. The inhibition of miR-381 in SMSC proliferation was also supported by down-regulating the expression of *Myf5* and *CCND1*, but up-regulating the expression of *CDKN1C* in caprine SMSCs. Of these genes, *Myf5* and *CCND1* were reported to positively regulate the proliferation of SMSCs [24], while *CDKN1C* inhibited the proliferation of SMSCs [25]. The inhibited effect of miR-381 on the viability and proliferation of cells has been reported in various human cell, especially for cancer cells, such as gastric cancer cells [26], lung cancer cells [27] and breast cancer cells [28]. miR-381 has also been reported to suppress proliferation in several types of smooth muscle cells [29,30].

To investigate the molecular mechanism by which miR-381 regulates the proliferation and differentiation of caprine SMSCs, we focused on pathways enriched by the predicted target genes of miR-381. Of these pathways, the Notch signaling pathway caught our attention as it can promote proliferation and maintain enough SMSCs to support postnatal muscle growth by preventing the premature differentiation of SMSCs [31,32,33]. It has been recognized that appropriate differentiation of SMSCs contributes to the growth of skeletal muscle, while premature and excessive myogenic differentiations lead to proliferation inhibition and depletion of SMSCs, resulting in the cessation of postnatal muscle growth [34]. In this study, miR-381 was found to target *JAG2*, which is a crucial member of the Notch signaling pathway. *JAG2* has been reported to prevent the number reduction and differentiation of SMSCs in chicken muscle development by inducing Notch activation [35]. Although the role of *JAG2* in SMSC proliferation was still unknown, another Notch ligand, *JAG1*, has been reported to promote proliferation of pig SMSCs by activating the Notch signaling pathway [3]. *PTEN* is a crucial upstream regulator of the Notch signaling pathway, and it was also found to be directly targeted by miR-381 in the study. The knock-out of *PTEN* prevented proliferation but caused premature differentiation of SMSCs in mice, finally resulting in the regeneration failure of skeletal muscle. Theoretically, *PTEN* deletion induced suppression of the Notch signaling by increasing Akt phosphorylation [36]. Taken together, it was concluded that miR-381 regulates proliferation and differentiation of caprine SMSCs by affecting the expression levels of *JAG2* and *PTEN* associated with the Notch signaling pathway.

In our studies, miR-381 was found to be down-regulated in muscle tissue of Liaoning cashmere goats with higher skeletal muscle mass compared to Ziwuling black goats. An increasing body of evidence has suggested that the increase in muscle fiber size in postnatal muscle requires sufficient SMSCs to provide additional myonuclei. On the contrary, skeletal muscle hypertrophy was diminished when SMSC proliferation was inhibited [1]. In this context, it was suggested that lower expression of miR-381 in Liaoning cashmere goats resulted in promoted proliferation and appropriate differentiation of SMSCs by regulating the Notch signaling pathway, finally leading to a higher muscle fiber size and skeletal muscle mass. A similar regulation mechanism has also been reposted by Hou et al. [6], who revealed that miR-34c inhibited proliferation and promoted the differentiation of porcine SMSCs by targeting *NOTCH1*. The regulation finally led to a decreased muscle fiber size and muscle mass in pigs [6].

## 4. Materials and Methods

### 4.1. Ethics Statement

All animal procedures in this study were approved by Animal Experiment Ethics Committee of Gansu Agricultural University (approval number GSAU-ETH-AST-2021-028).

### 4.2. Isolation, Identification and Myogenic Differentiation of Caprine SMSCs

Caprine SMSCs were primarily isolated from *Longissimus dorsi* muscle tissue collected from the area between the 12th and 13th ribs on the left carcass of a healthy one-month-old Ziwuling black ram, using the methods described by Ling et al. [14] and Sui et al. [9]. Briefly, after removing visible fascia, connective tissue and adipose tissue, the muscle samples were cut into 0.5–1.0 mm^3^ pieces. Subsequently, they were digested with 0.1% collagenase I (Solarbio, Beijing, China) at 37 °C for 50 min and then treated using 0.25% trypsin (Hyclone, Logan, UT, USA) at 37 °C for 15 min. The digestive fluid was filtered and cells were collected. After being washed two times with phosphate buffer solution (PBS), the cells collected were precipitated and then resuspended using growth medium containing 80% DMEM-F/12 (Hyclone, Logan, UT, USA) and 20% fetal bovine serum (Invigentech, Irvine, CA, USA). Finally, the cells were cultured at 37 °C with 5% CO_2_ and then purified three times using a differential adhesion method [9,14]; every two hours, the cell suspension was re-seeded in a new culture dish (Corning, NY, USA) for purification.

The purity of cultured caprine SMSCs was checked using a immunofluorescence staining analysis [14]. Briefly, purified cells cultured in 24-well plates were fixed in 4% formaldehyde for 20 min at 25 °C. Subsequently, the cells were permeabilized by 0.5% Triton X-100 for 20 min and then blocked with 5% BSA (Solarbio, Beijing, China) for 30 min at 25 °C. After incubation with rabbit anti-Pax7 primary antibody (1:300, Absin, Shanghai, China) at 4 °C overnight, the cells were then incubated with the goat anti-rabbit IgG FITC conjugated secondary antibody (1:500, Boster, Wuhan, China) at 37 °C for 2 h. Finally, the nuclei were stained with Hoechst 33258 (Solarbio, Beijing, China) for 10 min and the cells were viewed using an IX73 inverted fluorescence microscope (Olympus, Tokyo, Japan).

When the confluence of SMSCs in 24-well plates was over 90%, the growth medium was replaced by differentiation medium containing 98% high-glucose DMEM (Hyclone, Logan, UT, USA) and 2% horse serum (Hyclone, Logan, UT, USA) to induce differentiation of SMSCs, and the entire differentiation process lasted for 8 days. An IX73 inverted fluorescence microscope (Olympus, Tokyo, Japan) was used to observe myotubes formed during myogenic differentiation. For investigating the differentiation potential of caprine SMSCs, total RNA was extracted from SMSCs on days 0, 2, 4, 6 and 8 after SMSC differentiation using a Trizol reagent kit (Invitrogen, Carlsbad, CA, USA). The purity of the extracted RNA was assessed by measuring both the ratio of the absorbance at 260 and 280 nm (A260/A280) and the ratio of the absorbance at 260 and 230 nm (A260/A230), using a Nanodrop 2000 (Thermo Scientific, Waltham, MA, USA). Only RNA samples with both an A260/A280 ratio of 1.9–2.1 and an A260/A230 ratio of 2.0–2.2, were then used to synthesize cDNA using a HiScript III 1st Strand cDNA Synthesis Kit (Vazyme, Nanjing, China). The expression levels of two myogenic differentiation marker genes, *MyoG* and *MyHC*, were detected in triplicate using the 2 × ChamQ SYBR qPCR Master system (Vazyme, Nanjing, China) by RT-qPCR. A 20 µL reaction system was used to perform RT-qPCR analysis, including 2.0 µL of the cDNA, 0.4 µL of each primer, 10 µL of SYBR qPCR master mix (Vazyme, Nanjing, China) and 7.2 µL of RNase-free water. The thermal profile included an initial denaturation of 30 s at 95 °C, followed by 45 cycles of 95 °C for 10 s, 60 °C for 34 s and 95 °C for 15 s, and finishing with 60 °C for 60 s. The caprine *TUBB* was used as an internal control [37], and the primer information of the genes was listed in Table S1. The 2^−ΔΔCt^ method was used to calculate the relative expression level.

### 4.3. The Effect of miR-381 on Caprine SMSC Differentiation

To investigate the effect of miR-381 on the differentiation of caprine SMSCs, the expression profile of miR-381 at different differentiation stages was constructed, and the effect of miR-381 on the expression levels of myogenic differentiation marker genes, the area of MyHC-positive myotubes and the myogenic index were also investigated. Firstly, RNA extracted from SMSCs on days 0, 2, 4, 6 and 8 after myogenic differentiation inducement was used to produce the cDNA by the miRNA 1st Strand cDNA Synthesis Kit (Accurate Biology, Hunan, China). Caprine *U6* was used as an internal reference (Table S1) [14]. The expression levels of miR-381 at different differentiation stages were detected using RT-qPCR.

Secondly, when the confluence of SMSCs in 24-well plates was over 90%, either 50 nmol L^−1^ miR-381 mimic and mimic NC or 100 nmol L^−1^ miR-381 inhibitor and inhibitor NC synthesized by RiboBio Ltd. (Guangzhou, China) were transfected into SMSCs using the INVI DNA & RNA Transfection Reagent^TM^ (Invigentech, Irvine, CA, USA). Subsequently, the same method described above was used to induce differentiation of SMSCs at 24 h after transfection. On day 6 after differentiation inducement, the effects of miR-381 on the expression levels of myogenic differentiation marker genes, the area of MyHC-positive myotubes and the myogenic index were investigated. Briefly, the RNA was extracted and the expression levels of *MyoG*, *MyHC* and *MEF2C* were detected using RT-qPCR when caprine *TUBB* was selected as a reference gene [37]. In addition, anti-MyHC immunofluorescence staining was used to mark myotubes formed using the method described above. The monoclonal mouse anti-MyHC antibody (R&D Systems, Minneapolis, MN, USA) and goat anti-mouse IgG (H+L) CY3-conjugated (Affinity, Melbourne, Australia) were used as a primary and secondary antibodies, respectively, in the immunofluorescence assay. The myotube areas and myogenic index were finally measured by Image-Pro Plus v7.0 (Media Cybernetics, Inc., Rockville, MD, USA). Meanwhile, the cells without transfection of anything were designated as the non-transfection group to indicate the normal growth status of the cells in anti-MyHC immunofluorescence staining assay.

### 4.4. The Effect of miR-381 on the Viability and Proliferation of Caprine SMSCs

When the confluence of SMSCs cultured in 24-well plates was over 70%, either 50 nmol L^−1^ miR-381 mimic, 100 nmol L^−1^ miR-381 inhibitor or their corresponding NC was transfected into SMSCs using the INVI DNA & RNA Transfection Reagent^TM^ (Invigentech, Irvine, CA, USA), and the viability and proliferation of SMSCs were then investigated based on the following methods. For the CCK8 assay, the blank control group (containing only growth medium and CCK8 solution, but without cells) and non-transfection group (containing cells with non-transfection of anything, growth medium and CCK8 solution) were designed to normalize the absorbance value of the transfection group including cells transfected with miR-381 mimic, mimic NC, miR-381 inhibitor or inhibitor NC. The calculation formula of cell viability for the transfection group was as follows: Cell viability = (A − B)/(A_0_ − B). (A, absorbance of transfection group; B, absorbance of blank control group; A_0_, absorbance of non-transfection group). After transfection for 46 h, 30 µL of CCK-8 solution (Invigentech, Irvine, CA, USA) was added to each well and then incubated for 2 h. The absorbance at 450 nm wavelength was measured using a Varioskan LUX (Thermo Fisher Scientific, Waltham, MA, USA).

Meanwhile, a Cell-Light™ Edu kit (Beyotime, Shanghai, China) was used to detect the rate of DNA synthesis. Briefly, after transfection for 45 h, each well had 100 mL of 50 mM EDU reagent (Beyotime, Shanghai, China) added and then the plates were incubated for 3 h. Three areas of fluorescence from the stained nucleus were observed by an IX73 inverted fluorescence microscope (Olympus, Tokyo, Japan), and the percentage of EDU-positive SMSCs in all SMSCs was then counted by Image-Pro Plus v7.0. Meanwhile, the cells without transfection of anything were designated as the non-transfection group to indicate the normal growth status of the cells in the EDU staining assay. In order to further verify the effect of miR-381 on the proliferation of SMSCs, the expression levels of proliferation marker genes *Myf5*, *CCND1* and *CDKN1C* were also detected when miR-381 mimic, miR-381 inhibitor or their NC was transfected into SMSCs for 48 h. The total RNA was isolated from SMSCs, and RT-qPCR analysis of the three genes was carried out. The caprine *ACTB* was used as an internal control [14]. The corresponding primer information is listed in Table S1.

### 4.5. Prediction and Validation of the Target Genes of miR-381

To investigate the molecular mechanism by which miR-381 regulates the activities of caprine SMSCs, Miranda v3.3a, Targetscan v7.0 and miReap v.0.2 were used to predict its target genes, and the prediction results from the three kinds of software were overlapped. The KEGG pathway of the target genes predicted for miR-381 was analyzed using KOBAS 3.0. The predicted target genes *JAG2* and *PTEN* associated with the Notch signaling pathway were selected for a dual luciferase reporter assay, given that the pathway plays a key role in SMSC activities [31,32,33].

For the dual luciferase reporter assay, the 3′UTR sequences of *JAG2* and *PTEN* containing the binding site for a seed sequence of miR-381 were amplified using PCR primers (Table S1). The sequences amplified were ligated into the pmiR-RB-Report™ vector (RiboBio, Guangzhou, China) to construct wild-type pmiR-RB-Report™ vectors using restriction endonucleases Not1 and Xhol (Promega, Madison, WI, USA). Meanwhile, mutant pmiR-RB-Report™ vectors of *JAG2* and *PTEN*-3′UTR were generated using a Mut Express II Fast Mutagenesis Kit (Vazyme, Nanjing, China). The wild-type or mutant-type vectors constructed were validated by Sanger sequencing. The wild-type or mutant-type vectors (1 µg) and miR-381 mimic (100 pmoL) or mimic-NC (100 pmoL) were co-transfected into HEK293 T cells cultured in the same growth medium containing 80% DMEM-F/12 (Hyclone, Logan, UT, USA) and 20% fetal bovine serum (Invigentech, Irvine, CA, USA) using the INVI DNA & RNA Transfection Reagent^TM^ (Invigentech, Irvine, CA, USA). The luciferase activities of *JAG2* and *PTEN* at 48 h after transfection were detected using the dual luciferase reporter assay system (Promega, Madison, WI, USA) by a Varioskan LUX (Thermo Fisher Scientific, Waltham, MA, USA).

To investigate the effect of miR-381 on the expression of the target genes, the miR-381 mimic, miR-381 inhibitor and the corresponding NC were transfected into SMSCs using the method described above. After transfection for 48 h, the expression levels of *JAG2* and *PTEN* were quantified using RT-qPCR. The caprine *ACTB* was used as an internal control [14] (Table S1).

### 4.6. Statistical Analysis

Statistical analysis was performed using SPSS v24.0 (IBM, Armonk, NY, USA), and data are presented as mean ± standard error of the mean for three replicates. The differences between the two groups were compared using the two-tailed independent *t*-test, with *p* < 0.05 taken as statistically significant.

## 5. Conclusions

In summary, miR-381 promoted myogenic differentiation of caprine SMSCs, and it inhibited the viability and proliferation of the cells by targeting *JAG2* and *PTEN*. This study provides a better understanding of the functions of miR-381 in skeletal muscle growth and development.

## Data Availability

The data presented in this study are available in the article and Supplementary Materials.

## Supplementary Materials

The following supporting information can be downloaded at: https://www.mdpi.com/article/10.3390/ijms232113587/s1, Figure S1: Isolation, identification and myogenic differentiation of caprine skeletal muscle satellite cells (SMSCs). Table S1: List of the primers used in this study.
