# Supplementary material for: MicroRNA-381 Regulates Proliferation and Differentiation of Caprine Skeletal Muscle Satellite Cells by Targeting PTEN and JAG2
Source: Int J Mol Sci. 2022 Nov 5;23(21):13587. doi: 10.3390/ijms232113587 (PMC9656929; doi:10.3390/ijms232113587)
Supplement: Supplementary file 1 [file ijms-23-13587-s001.zip › ijms-1996034-supplementary.pdf]

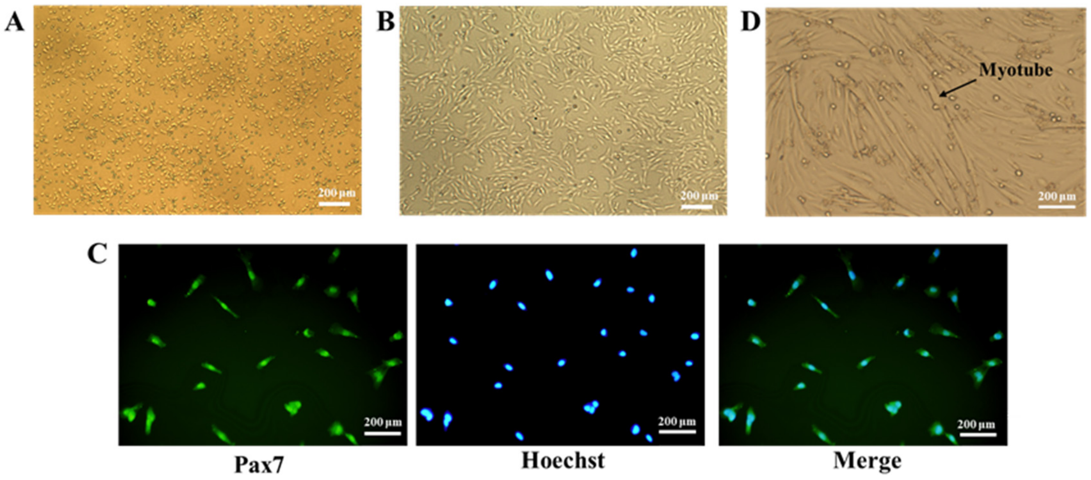

Figure S1. Isolation, identification and myogenic differentiation of caprine skeletal muscle satellite cells (SMSCs). The morphology of isolated SMSCs cultured in growth medium for 48 h (**A**) and 96 h (**B**). (**C**) Identification of isolated SMSCs using immunofluorescence staining of Pax7. (**D**) SMSCs were differentiated into myotubes on day 6 after differentiation initiation.

Table S1. List of the primers used in this study.

miRNA/gene	Forward (5' to 3')	Reverse (5' to 3')
miR-381	TATACAAGGGCAAGCTCTCTGT	mRQ 3′ primer[1]
U6	GGAACGATACAGAGAAGATTAGC	TGGAACGCTTCACGAATTTGCG
ACTB	AGCCTTCCTTCCTGGGCATGGA	GGACAGCACCGTGTTGGCGTAA
Myf5	CACAACCAACCCTAACCA	TTGATCCGATCCACTATG
TUBB	AGCGTATCTCAGAGCAGTTC	AATCCTCTTCCTCTTCTGCG
CCND1	CGTCCATGCGGAAGATCGT	ACAGGAAGCGGTCCAGGTAGT
CDKN1C	CAGCCAGAGCATTGGCAATG	CTGTCCACCTCGGTCCACT
MyoG	CGTGGGCGTGTAAGGTGT	GGCGCTCTATGTACTGGATGG
MyHC	CCACATCTTCTCCATCTCTG	GGTTCCTCCTTCTTCTTCTC
MEF2C	ATCCTGATGCAGACGATTCAG	GGTGGAACAGCACACAATCTT
PTEN-wild[2]	CCGCTCGAGTGAGATGAAAGAGACTGATTTTCC	AAATATGCGGCCGCTGGAAAGGGCTCTTCAGTACA
PTEN-mut[3]	GTTGAACATAATTCATGTATATATTTTCTTTAAATGGTGAA	CATGAATTATGTTCAACAAAAACAGGATTGTATAGTCACTGA
PTEN-CDS[4]	CGACGGGAAGACAAGTTCAT	AGGTTTCCTCTGGTCCTGGT
JAG2-wild[5]	CCGCTCGAGGCCCGGGCGGCCGGCCCGCT	AAATATGCGGCCGCACCACAGGAGCCCCCGGCAGC
JAG2-mut[6]	CTGTAGCGAACATAAATTATTCGGTAACTGTCAGGCG	ATTTATGTTCGCTACAGAAACGTAGAAAATAAACATTTG
JAG2-CDS[7]	CGCCAATGAGTGTGAAGGGA	TCGTTGGCGTTGATGTGGC

[1] A universal down-stream primer used for RT-qPCR analysis of miRNA.

[2, 5] Primers used to amplify 3'UTR sequence of *PTNE* and *JAG2* in wild-type vectors constructed, respectively.

[3, 6] Primers used to amplify complementarily mutant sequence to 3'UTR sequence of *PTNE* and *JAG2* in mutant-type vectors constructed, respectively.

[4, 7] Primers used to amplify the coding sequence of *PTNE* and *JAG2*, respectively.
